# Clinical Evaluation of the Osteoporotic Fracture Treatment Score (OF-Score): Results of the Evaluation of the Osteoporotic Fracture Classification, Treatment Score and Therapy Recommendations (EOFTT) Study

**DOI:** 10.1177/21925682221148133

**Published:** 2023-04-21

**Authors:** Bernhard W. Ullrich, Klaus John Schnake, Philipp Schenk, Sebastian Katscher, Martin Bäumlein, Volker Zimmermann, Falko Schwarz, Gregor Schmeiser, Michael Scherer, Michael Müller, Kai Sprengel, Katja Liepold, Simon Schramm, Hagen-Christopher Baron, Holger Siekmann, Alexander Franck, Max J. Scheyerer, Seyma Kirtas, Ulrich J. A. Spiegl, Georg Osterhoff, Working Group Osteoporotic Fractures of the Spine Section of the German Society of Orthopaedics and Trauma

**Affiliations:** 1 Department of Trauma and Reconstructive Surgery, 39781 BG Clinic Bergmannstrost Halle, Germany; 2 Department of Trauma, Hand and Reconstructive Surgery, 61061 Jena University Hospital, Friedrich Schiller University, Jena, Germany; 3 Center for Spinal and Scoliosis Surgery, 40658 Waldkrankenhaus Erlangen, Germany; 4 Department of Orthopedics and Traumatology, Paracelsus Private Medical University Nuremberg, Germany; 5 Department of Science, Research and Education, BG Clinic Bergmannstrost Halle, Germany; 6 Department of Spine Surgery and Neurotraumatology, Sana Klinikum Borna, Borna, Germany; 7 Center for Orthopaedics and Trauma Surgery, University Hospital Giessen-Marburg, Germany; 8 Department of Trauma and Orthopedic Surgery, Klinikum Traunstein, Germany; 9 Department of Neurosurgery, 39065 Jena University Hospital, Friedrich Schiller University, Germany; 10 Department of Spine Surgery, Schoen-Clinic Hamburg Eilbek, Hamburg; 11 Medical Faculty Technical University of Munich, Germany; 12 Department of Orthopedic and Trauma Surgery, University Medical Center Schleswig-Holstein, Campus Kiel, Germany; 13 Hirslanden Clinic St Anna, University of Lucerne, Lucerne, Switzerland; Department of Trauma, University Hospital Zurich (USZ), University of Zurich (UZH), Switzerland; 14 Department of Spine Surgery, Thuringia Clinic “Georgius Agricola” Saalfeld, Teaching Hospital of the University of Jena, Germany; 15 Department of Trauma and Orthopedic Surgery, University Hospital Erlangen, Germany; 16 Department for Paraplegia and Spine Surgery, BG Clinic Tuebingen, Tuebingen, Germany; 17 Clinic of Trauma- Hand- and Reconstruction Surgery, AMEOS-Clinic Halberstadt, Germany; 18 Department of Trauma Surgery and Orthopedics, Regiomed Clinical Center Coburg, Germany; 19 Faculty of Medicine and University Hospital Cologne, Department of Orthopaedic and Traumatology, University of Cologne, Germany; 20 Department of Orthopaedics, Trauma and Plastic Surgery, University Hospital Leipzig, Germany

**Keywords:** osteoporosis, treatment, vertebral fracture, operative, OF classification

## Abstract

**Study Design:**

Multicenter prospective cohort study.

**Objective:**

The study aims to validate the recently developed OF score for treatment decisions in patients with osteoporotic vertebral compression fractures (OVCF).

**Methods:**

This is a prospective multicenter cohort study (EOFTT) in 17 spine centers. All consecutive patients with OVCF were included. The decision for conservative or surgical therapy was made by the treating physician independent of the OF score recommendation. Final decisions were compared to the recommendations given by the OF score. Outcome parameters were complications, Visual Analogue Scale, Oswestry Disability Questionnaire, Timed Up & Go test, EQ-5D 5 L, and Barthel Index.

**Results:**

In total, 518 patients (75.3% female, age 75 ± 10) years were included. 344 (66%) patients received surgical treatment. 71% of patients were treated following the score recommendations. For an OF score cut-off value of 6.5, the sensitivity and specificity to predict actual treatment were 60% and 68% (AUC .684, *P* < .001). During hospitalization overall 76 (14.7%) complications occurred. The mean follow-up rate and time were 92% and 5 ± 3.5 months, respectively. While all patients in the study cohort improved in clinical outcome parameters, the effect size was significantly less in the patients not treated in line with the OF score’s recommendation. Eight (3%) patients needed revision surgery.

**Conclusions:**

Patients treated according to the OF score’s recommendations showed favorable short-term clinical results. Noncompliance with the score resulted in more pain and impaired functional outcome and quality of life. The OF score is a reliable and save tool to aid treatment decision in OVCF.

## Introduction

Osteoporotic vertebral compression fractures (OVCF) have become a relevant issue for health care systems. In 2019, the incidence in Germany was 255/100′000 for lumbar and 137/100′000 for thoracic fractures among individuals older than 70 years. This represents an increase of 21% for lumbar and of 32% for thoracic vertebral fractures over the last 10 years.^1^

Some authors suggest conservative therapy in acute OVCF and surgical therapy in case of painful nonunion and kyphosis only.^2^ Although conservative therapy is successful in most cases, clear indications for treatment of acute OVCF are still missing.^3^

The Working Group “Osteoporotic Fractures” (AG-OF) of the Spine Section of the German Society of Orthopaedics and Trauma (DGOU) has developed a new reliable and reproducible classification system for osteoporotic fractures (OF classification).^4^ The development process of the OF classification followed the concept for validation of fracture classifications according to Audigé et al^5^ In the next step a score for therapeutic decision making based on the OF classification was developed^6^ (Table 1). To evaluate the score prospectively, the AG-OF designed the “Evaluation of the Osteoporotic Fracture classification, Treatment score and Therapy recommendations” (*E*OF*TT*) study.

**Table 1.** Osteoporotic Vertebral Fracture Score (OF Score).

Parameter	Grade	Points
OF-classification (morphology)	1-5	2-10
Severity of osteoporosis	T-score <3	1
Deformity progression	Yes/No	1/-1
Pain (under adequate analgesia)	VAS ≥4/<4	1/-1
Fracture related neurological deficit	Yes	2
Able to mobilize without help	Yes/No	-1/1
Health status	ASA >3, BMI <20 kg/m^2^, nursing case, anticoagulation	Each parameter -1; maximum -2

Abbreviations: ASA, American society of anesthesiologists risk classification; BMI, Body mass index; VAS, Visual analogue scale for pain. The OF classification grade is doubled and summarized with the results of the items on osteoporosis, deformity progression, pain, neurological deficits (complete or incomplete damage to the central or peripheral nervous system caused by the index fracture in the sense of radiculopathy, myelopathy and or cauda equine syndrome), mobility, and general health state. 0 points are given if a parameter is unknown or not determinable. For 0-5 points conservative, for 6 points indifferent and for >6 points surgical recommendation is given.

## Materials and Methods

A prospective multicenter observational study was conducted. Approval from institutional or regional ethical committees was obtained by all participating centers and all patients gave written informed consent to participate in the study. Data of patients with OVCF were collect prospectively in 17 spine centers in Germany and Switzerland. Included were patients older than 18 years hospitalized for OVCF, either atraumatic or due to a low energy trauma, and with proven osteoporosis following national and international recommendations.^7^ In cases where more than one fracture was present, the fracture that was the most severe according to the OF classification was used for the evaluation and treatment decision. Treatment followed the standards of each center and the final decision was made by the individual surgeon treating the patient independently from the study.

A standardized pseudonymized Case Report Form (CRF) was used for data collection. On admission, the OF-score was calculated as depicted in Table 1. The score was calculated on a daily base until final decision of the therapy was made.

For clinical evaluation, the type of treatment (conservative or surgical) was recorded. Conservative treatment included prescription of analgesics up to level 3 of the WHO ladder scheme, mobilization training, physiotherapy, and exercises. The use of orthoses was optional. In case of a surgical decision, it was recommended to follow the treatment recommendations of the AG-OF published by Blattert et al^6^ The following scores were obtained at time of treatment decision and at final follow-up (FU): Oswestry Disability Index (ODI), visual analogue scale subjective health state form EQ5D (VAS-EQ5D), EQ5D-5L index value, Barthel, Timed up and go test (TuG) and Pain at visual analogue scale (VAS-P). The EQ5D-5L was calculated using the data set for German index values. The higher the value, the better the health status up to a maximum of 1. The reported subjective health status in the EQ5D (VAS-EQ5D) was chosen using a visual analogue scale (VAS) with the limits of 0 (zero) and 100, where 100 reflects the best and 0 the worst health status. The TuG measures relevant mobility impairment by measuring the patient’s time needed for stand up on a chair with armrests, walk a distance of 3 m, turn around walk back and sit down again. Four follow up visits were scheduled (6 and 12 weeks and 6 and 12 months). If not all 4 visits were made the last one was used for data analysis.

Depending on performed treatment and the OF score recommendation, the patients were divided in 6 groups. The first group’s recommendation was conservative and therapy was conservative (conservative-compliant); the second group’s recommendation was conservative, but the therapy was surgical (conservative-noncompliant); the third group’s recommendation was indifferent (indifferent-conservative) and received conservative therapy; the fourth group’s recommendation was also indifferent (indifferent-surgical), but received surgical therapy; the fifth group’s recommendation was surgical and therapy was surgical (surgical-compliant); and the sixth group’s recommendation was surgical, but received conservative therapy (surgical-noncompliant).

### Statistical Analysis

The positive and negative predictive values (PPV and NPV) of the OF score for performed therapy were calculated. ROC and Youden-Index analysis for cut off value and its sensitivity and specificity calculating the OF-score threshold for surgical therapy recommendation were performed and the area under the curve (AUC) was calculated.

For visual and statistical evaluation, the data of the clinical parameters over group affiliation (compliant, indifferent, noncompliant) were presented graphically as mean values and 95% confidence intervals (CI). For pairwise comparison, the error bars of the 95% CI are used. An overlap of the lower and upper limits of the 95% CI indicates that there exists no difference in mean. If the error bars do not overlap, there is a difference in means at a probability of error of 5%.

Separate T-tests were conducted for detection of mean differences of clinical outcome at the hospitalization/day of treatment decision between the 2 groups patients for whom the score had recommended conservative treatment and who were treated surgically (conservative-incompliant) and patients for whom surgical treatment was recommended and who were treated conservatively (surgical-noncompliant). T-Tests were performed to find differences in means between the both groups with indifferent recommendation.

Differences in occurrence of adjacent fracture at FU between surgically and conservatively treated patients were checked with Fisher exact Chi^2^ Test.

For statistical analysis, the IBM software SPSS V.27 for Windows (IBM Corp. Released 2020. Armonk, NY: IBM Corp) was used. The level of significance was set to *P* = .05.

## Results

In total, 518 patients (390 female, 75.3%) with an age of 75 ± 10 years (range, 41 to 97 years) and 518 OVCF were included in the study. The mean age did not differ between males and females (*P* = .150). Two-thirds of the patients (n = 338) reported a trauma while 180 could not remember any trauma.

65% of the fractures were located at the thoracolumbar junction (Th11-L2). OF 3 was the most common fracture type (n = 215, 42%). OF 2 and OF 4 types occurred in 127 cases (26%) and 139 cases (27%) respectively. OF 1 and OF 5 fractures were present in 1% (n = 4) and 4% (n = 23), respectively.

With regard to the mobility before sustaining the OVCF, 77% of all patients had been fully mobile and 22% had already used walking aids. In one case each, the patient was bedridden or just able to stand previous to the fracture.

In 86% of the cases, the OF score clearly recommended either conservative or surgical therapy. Seventy-four cases (14%) had an OF score value of 6 and therefore treatment was at the discretion of the treating physicians. Of these patients with an indifferent score recommendation, 77% were treated surgically and 23% received conservative therapy.

In 315 cases (71%) the performed therapy was in concordance with the recommendation of the OF score. Thus, in 29% of the cases, the physicians did not follow the OF score recommendation (Table 2).

**Table 2.** Number of Patients with Regard to the Recommended and the Finally Performed Therapy.

		Of Score Recommendation			Total
		Conservative	Indifferent	Surgical	
Performed therapy	Surgical	82	56	206	344
	Conservative	109	19	46	174
Total		191	75	252	518

Overall, 344 (66%) fractures were treated surgically. The remaining 174 (33%) fractures were treated conservatively, using physiotherapy and/or orthoses.

For an OF score cut-off value of 6.5, the sensitivity and specificity to predict actual treatment were 60% and 68%, respectively (AUC .684, *P* < .001).

Clinical outcome could be assessed for 478 patients (92.3%) with a mean follow up of 5 ± 3.5 months, of these 319 had undergone surgical and 159 conservative treatment. Regardless of the therapy chosen, each clinical outcome improved significantly (all *P* < .001) during the follow up.

At the time of treatment decision, patients with an OF score recommendation for conservative therapy who received surgical treatment (conservative-noncompliant) had significantly higher pain values (VAS-P: *P* < .001), higher ODI scores (*P* = .017), lower EQ5D-5L index scores (*P* = .002), and worse subjectively reported health status (VAS-EQ5D: *P* < .001) compared to conservative recommended and conservative treated (conservative-compliant) patients. No differences were found for TuG (*P* = .270) and Barthel score (*P* = .194).

Patients for whom the score recommended surgery who were treated conservatively had less pain (VAS-P: P < .001), lower Barthel-scores (P < .001), better ODI values (P < .001), better EQ5D index values, and higher VAS-EQ5D (P = .001, P < .001) at the time of treatment decision. The TuG indicated sig. better mobility (P = .021) for these patients.

Surgically treated patients with an OF score of 6 (indeterminate) suffered from significant higher VAS-P values compared to the conservatively treated patients with the same score (*P* = .001). No sig. differences could be found for TuG (*P* = .170), Barthel-score (*P* = .176), ODI (.842), and for both EQ5D outcome scores (VAS-EQ5D: *P* = .303, EQ5D index value: *P* = .931), respectively.

The magnitude of change in clinical outcome parameters are given as effect sizes in Table 3 differentiated for OF-score recommendation and finally performed therapy (compliant or noncompliant).

**Table 3.** Change of Outcome Parameter as Effect Size (Cohen´s d) From Day of Treatment Decision to Follow Up Examination for Visual Analogue Scale Pain (VAS-P) Timed Up and Go Test (TuG) Oswestry Disability Index (ODI), Barthel, EQ5D-5L Index Value and visual analogue scale EQ5D-5l Self Reported Health Status (VAS-EQ5D).

Of score	PerformedTherapy	VAS-P	TuG	ODI	Barthel	EQ5D-5LIndex Value	VAS-EQ5D
<6	Conservative:	0.63	0.63	1.12	0.69	0.72	0.54
	Surgical:	1.13	0.41	1.18	0.64	1.00	0.78
6	Conservative:	1.66	1.01	2.10	0.58	1.92	0.92
	Surgical:	1.61	0.56	1.73	1.36	1.61	1.17
>6	Conservative	0.66	0.56	1.12	0.67	1.08	0.60
	Surgical	1.37	0.80	1.70	1.00	1.48	1.13

During hospitalization overall 66 (13%) complications 9 (5%) in conservatively and 57 (17%) in the surgically treated group (*P* = .001) where recorded. Individual patients also showed more than one complication. Two deep wound infections and 3 superficial wound healing disorders occurred. Revision surgery due to this during hospitalization was necessary in 4 cases (1%). In 2 other cases, revision surgery was necessary due to non-infectious implant complications. Urinary tract infections were the most common complication in the conservative (n = 5 (3%)) and surgical (n = 29 (8%)) group.

In 12 (3%) of the 478 patients who were seen at follow up examinations, a change from conservative to surgical treatment had been necessary (8 (7%) in the conservative-compliant, 3 (7%) in the surgical-noncompliant and one (5%) in the indifferent-conservative group).

Symptomatic and asymptomatic adjacent level fractures were seen in 24 surgically treated cases (8%) and in 6 conservatively treated patients (4%) *P* = .160.

## Discussion

Very few treatment recommendations regarding OVCF exist and therapy remains controversial (2, 3, 6). In contrast, for bone healthy patients with thoracolumbar fractures the Thoracolumbar AOSpine Injury Score exists.^8,9^ For other conditions, such as spinal tumors, the Spinal Neoplastic Instability Score, was developed through a structured interactive process.^10^ So, the AG-OF decided to close this knowledge gap and has developed the OF score (6) to aid treatment decisions based on the recently developed OF classification (4). The score acknowledges the specific clinical and radiological peculiarities of the generally elderly patients with OVCF. In a prospective multicenter study, 518 patients in 17 spine centers could be included of whom 92% were available for follow-up.

The treatment recommendation was either conservative (<6 pts), indifferent (6 pts) or surgical (>6 pts) but the final treatment modality was at the surgeons’ discretion.

The score recommended for either surgical or conservative therapy in 86%, underlining the fact that OF score is able to set a clear recommendation in the majority of cases. ROC analysis yielded sensitivity and specificity optimized cut off value for the OF score of 6.5 pts. This confirms the current threshold of 7 for surgical recommendation.

**Figure 1.**
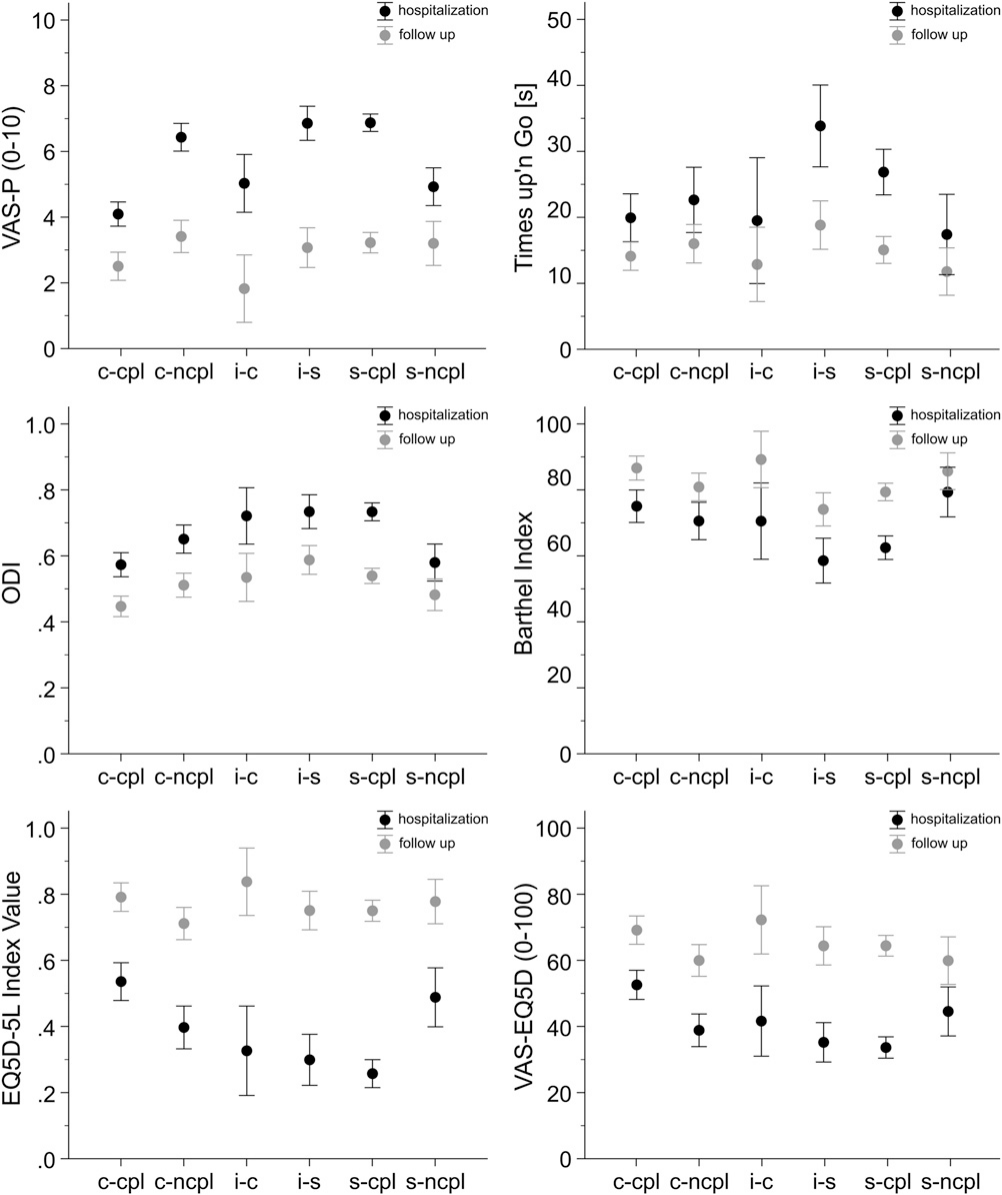
Clinical assessments at day of treatment decision and final follow up. VAS-Pain, Timed up’n Go Test, Oswestry Disability Index (ODI), Barthel Index, EQ5D 5L Index Value and EQ5D self reported health status. Data are given as mean ± 0.95 confidence intervals. The clinical assessments are given in 6 groups, corresponding to the OF-score recommendation: conservative recommended-compliant treated (c-cpl), conservative recommended-noncompliant treated (c-ncpl), indifferent recommendation-conservatively treated (i-c), indifferent recommendation-surgically treated (i-s), surgically recommended-compliant treated (s-cpl), surgically recommended-noncompliant treated (s-ncpl).

The majority (71%) of patients were treated following the score recommendation and showed relevant improvement of the functional parameters. While all patients in the study cohort improved in clinical outcome parameters (Figure 1), the effect size was less in the noncompliant groups.

In comparison to the conservative group, there was a higher beneficial effect size in clinical parameters notable for patients with surgical recommendation and surgical therapy. This indicates that score-conform surgical therapy is very likely to lead to a successful outcome.

Of note, surgical and conservative treatment yielded very similar effect sizes in the indifferent group (6 pts) underlining the relevance of such a “grey zone” where both treatment options are possible.

Of the patients who were treated conservatively as recommended by the OF score, 8 (7%) patients failed in the later course and had to be operated. Failure of conservative treatment was due to radiological and clinical deterioration and increase in the OF score accordingly. It must be emphasized that in case of secondary deformity, fracture upgrade, increasing pain, or reduced mobility the score may change. Thus, conservatively treated patients should undergo frequent reevaluation – radiologically, clinically and by the OF score.

Twenty-nine percent of the patients were not treated according to the recommendation of the OF score. A detailed analysis revealed that patients with surgical recommendation but conservative therapy were clinically significantly less limited (VAS-P <6, ODI <.7, Barthel >70, EQ5D-Index value >.4, and VAS-EQ5D >45).

In contrast, patients of the noncompliant group with conservative recommendation but surgical therapy presented with worse subjective parameters (VAS-P ≥6 and VAS-EQ5D <45).

In the indifferent group with 6 points in the OF score only VAS-P (≥6) was significantly different between surgically and conservatively treated patients.

As a consequence, the cut-off value of 4 for the VAS has to be critically discussed and possibly adjusted.

Overall 66 (13%) complications occurred during hospitalization. Early revision surgery was necessary in 6 (2%) of 344 surgically treated patients only. This seems to be an acceptable rate in relation to the literature.^11^ Adjacent level fractures occurred with 8% in the surgically treated group which is a lower rate than in other studies^12^ 1 reason could be the shorter follow up interval in our study.

This study has several limitations. Even though 92% of the patients were available for follow-up, the follow-up periods were inconsistent and rather short. Especially the rate of adjacent fractures may differ among the different groups with longer observation periods. Limited information was available on the reasons why in some cases a treatment was chosen that was not congruent with the recommendation of the OF score and the majority of patients included underwent surgical treatment as only inpatients were included.

## Conclusion

This study evaluated the OF score for treatment decisions in OVCF. Patients treated according to the score’s recommendations showed favorable short-term clinical results. Noncompliance with the score’s recommendations was associated with more pain and impaired functional outcome and quality of life. The OF score is a reliable and save tool to aid treatment decision in OVCF. Further adjustment of the score may increase compliance in the future.
